# Posttransplant Lymphoproliferative Disorder (PTLD): 24 Years of Experience at a Referral Center in São Paulo, Brazil. Can Differences in Prevalence and Subtype of EBV Infection in the Population Influence the Results? A Retrospective Cohort Study

**DOI:** 10.1155/joot/6468943

**Published:** 2025-12-28

**Authors:** Juliano Córdova Vargas, Ricardo Helman, Marcelino de Souza Durão, Erika Ferraz de Arruda, José Eduardo Afonso, Rafael Medeiros Carraro, Lilian Amorim Curvelo, Guilherme Eduardo Gonçalves Felga, Celso Eduardo Lourenço Matielo, Patrícia Holanda Almeida, Denise Pasqualin, Renata Stanzione, Carolina Perrone, Guilherme Perini, Nelson Hamerschlak

**Affiliations:** ^1^ Haematology Department, Higienópolis Samaritan Hospital, São Paulo, São Paulo, Brazil; ^2^ Haematology Department, Hospital Israelita Albert Einstein, São Paulo, São Paulo, Brazil, einstein.br; ^3^ School of Medicine, São Camilo University Center, São Paulo, São Paulo, Brazil; ^4^ Kidney Transplant Unit, Hospital Israelita Albert Einstein, São Paulo, São Paulo, Brazil, einstein.br; ^5^ Nephrology Division, Universidade Federal de São Paulo (UNIFESP), São Paulo, São Paulo, Brazil, unifesp.br; ^6^ Lung Transplant Unit, Hospital Israelita Albert Einstein, São Paulo, São Paulo, Brazil, einstein.br; ^7^ Hepatology Transplant Unit, Hospital Israelita Albert Einstein, São Paulo, São Paulo, Brazil, einstein.br; ^8^ Pathology Department, Hospital Israelita Albert Einstein, São Paulo, São Paulo, Brazil, einstein.br

**Keywords:** EBV subtype, lung transplantation, posttransplant lymphoproliferative disorder, PTLD

## Abstract

**Objectives:**

To retrospectively review all cases of posttransplant lymphoproliferative disorder (PTLD) in a large Brazilian transplant center, describing patients’ clinical, virological, and histopathological profiles and treatment strategies and prognostic factors.

**Methods:**

This retrospective cohort study was conducted between January 2000 and June 2024. Adult patients with confirmed PTLD following solid‐organ or bone marrow transplant were included. Patients with other systemic cancers or on concurrent chemotherapy/radiotherapy were excluded. Clinical characteristics, PTLD prevalence, histopathology, and survival were assessed.

**Results:**

Thirty‐eight cases of PTLD were identified in the 5928 transplant patients (0.6%). Incidence was highest in lung recipients (31%). Median time to PTLD onset was 42 months. EBV DNA was detectable in 54.8% of cases. Monomorphic PTLD was the most common (89.5%), primarily in non‐Hodgkin lymphomas (91.2%). Immunotherapy (anti‐CD20) and immunosuppression reduction were standard initial treatments. R‐CHOP and rituximab monotherapy were the main first‐line regimens. Age and treatment response significantly influenced overall survival. Mortality was 42%, mainly due to infections and disease progression.

**Conclusions:**

Despite the higher prevalence of EBV in Brazil, PTLD patterns and incidence were consistent with those found in developed countries. The strong association with lung transplants mirrors global data. Local EBV subtype characteristics and host immunogenetic factors warrant further investigation.

## 1. Introduction

Posttransplant lymphoproliferative disorders (PTLDs) are lymphoid and/or plasmacytic proliferations that occur in patients receiving chronic immunosuppression following solid‐organ transplantation or allogeneic hematopoietic cell transplantation (HCT) [1, 2]. Oncogenic viruses such as HIV, HTLV‐1, and Epstein–Barr virus (EBV) facilitate the malignant transformation of lymphoid cells. In particular, EBV, a ubiquitous herpesvirus infecting over 90% of the global population, is strongly associated with PTLDs. This condition is a severe and potentially fatal complication following solid‐organ or hematopoietic stem cell transplantation. Mortality associated with monomorphic PTLD has been reported to be as high as 80%. T‐cell lymphomas, in particular, carry an extremely poor prognosis [3, 4]. Immunosuppression plays a pivotal role in the pathogenesis of lymphoproliferative disorders. This involves the immune surveillance of EBV Genotype 1, a virus prevalent in Brazil and much of South America, which has greater lymphomagenic potential through B‐cell immortalization [1, 2]. Moreover, the LMP1 oncogene and its polymorphisms, commonly expressed in this population, may be linked to more aggressive disease. EBV‐negative disease is a rare form of PTLD and has been documented in up to 30% of cases in some series [3, 4].

The risk of PTLD correlates with the level and duration of immunosuppression, primary EBV infection, and the type of graft [5, 6]. Unlike developed countries, data from Latin America, particularly Brazil, remain sparse, despite the distinct epidemiology of EBV in this region. Immunosuppression in HIV‐infected patients is also strongly linked to cancer. While the introduction of antiretroviral therapy drastically reduced the incidence of most AIDS‐defining cancers, lymphoma rates did not decline as significantly. Lymphoma has become the most prevalent cancer in HIV patients, accounting for over 50% of AIDS‐related illnesses, and is now the leading cause of death in this population [7, 8].

This study presents a comprehensive 24‐year analysis of PTLD cases in a large Brazilian transplant center. In this paper, the clinical, virological, and histopathological profiles of affected patients are described, as well as the treatment strategies used and the prognostic factors that impacted survival. In addition, PTLD is compared to cases of HIV‐associated lymphomas, highlighting the immunological parallels between these two populations. There is a paucity of information regarding the clinical profile of patients with PTLD in Brazil. This study underscores the importance of integrating local epidemiologic data with global evidence to refine future PTLD management strategies.

## 2. Methods and Materials

This retrospective and single‐center study was conducted at the Hospital Israelita Albert Einstein (HIAE) in São Paulo, Brazil. The hospital’s internal review board approved the study protocol, and eligible patients were asked to read and sign the informed consent form during a face‐to‐face interview with one of the researchers (Juliano Córdova Vargas or Ricardo Helman). Participants consented to their clinical data being retrieved from medical records and used in this study. For patients who were no longer being followed up at the institute, the form was delivered electronically via the RedCap platform. Informed consent was waived in cases of death or after three attempts to contact the patient or a family member, made once a week at different times of the day, proved ineffective. This manuscript is reported according to the STROBE checklist. Data will not be shared publicly due to the possibility of patient identification.

Patients over 18 years of age, suffering from lymphoma, and with a confirmed diagnosis of PTLD made between 1 January 2000 and 1 June 2024, were included. The study used a convenience sample of all consecutive patients admitted during this period, with no previous calculation of sample size. Patients who had another systemic neoplasm or were undergoing concomitant chemotherapy and/or radiotherapy were excluded from the study. Demographic, clinical, and epidemiological data; laboratory results; and data on cancer staging, histopathology, treatment, treatment response, relapses, and retreatment were extracted from medical records using a form specifically created for this study and registered in a Microsoft Excel spreadsheet accessible to the entire research team. One author (Juliano Córdova Vargas) double‐checked data entry.

Patients’ data registered at the time of PTLD diagnosis included the following: age, sex, organ transplanted, histopathological classification, time from organ transplant until PTLD diagnosis, stage, symptoms, extranodal involvement, and immunosuppression regimen. Laboratory records consisted of hemoglobin levels, lactate dehydrogenase (LDH), albumin, beta‐2 microglobulin, and polymerase chain reaction (PCR) for EBV DNA.

The diagnosis of PTLD was confirmed from the results of tissue biopsy, which was, as per hospital routine, reviewed by two experienced pathologists (not any of the authors), with a consensus being reached in all cases. The diagnosis was then registered for the purpose of this study in accordance with the 2017 World Health Organization (WHO) International Classification of Lymphomas [9, 10] into polymorphic, monomorphic, and hyperplastic subtypes. CD20 immunohistochemistry and PCR for EBV DNA detection were performed on paraffin‐embedded tissue specimens. Staging was performed using oncological positron emission tomography–computed tomography (PET‐CT), and the lymphoma was classified as Ann Arbor Stages I–IV.

The presence of B symptoms was recorded. Response to treatment or disease progression was determined using PET‐CT according to the Deauville score and the Cheson et al. criteria [11].

The immunosuppressive regimens used by the transplant recipients included calcineurin inhibitors as monotherapy or in combinations with purine analogs or as triple therapy including glucocorticoids. The patients initially underwent immunosuppression reduction, followed or not by regimens of rituximab monotherapy or polychemotherapy.

In the statistical analysis, factors that could have affected patient outcomes and survival were evaluated. The categorical variables available for all the patients with PTLD were evaluated using absolute and relative frequencies, with associations assessed using the chi‐square test and Fisher’s exact test or the likelihood ratio test. The continuous variables were described using means and standard deviations (SDs) or medians and interquartile range (IQR) and compared using Student’s *t*‐test and the Mann–Whitney test [12].

Survival times following PTLD diagnosis were compared according to different qualitative characteristics using log‐rank tests or bivariate Cox regression, with 95% confidence intervals (95% CIs) calculated to estimate mortality risks for each variable evaluated. A final multivariate Cox regression model for survival included variables with *p* values < 0.10 in the bivariate analysis. The data analysis and tabulation were conducted using SPSS, Version 20.0 for Windows, and Excel 2003. Significance level was set at 5% for the entire statistical analysis.

## 3. Results

**Figure 1:**
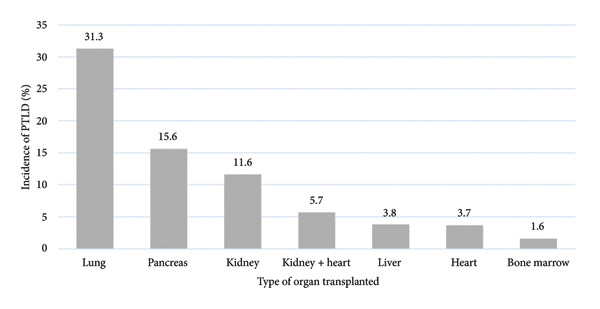
Incidence of PTLD according to the type of organ transplanted between 1 January 2000 and 1 June 2024.

During the study period, 5928 organ transplants were performed at the HIAE. A breakdown of the organs transplanted is provided in Table 1. Of these patients, 38 cases of PTLD (0.6%) were registered. As shown in Figure 1, the highest incidence (31%) was among lung transplant recipients. The onset of PTLD occurred after a median of 42 months following organ transplant.

**Table 1:** Characteristics of the patients diagnosed with posttransplant lymphoproliferative disorder (PTLD).

Characteristic	*n*	%	Total number of transplant recipients	Incidence of PTLD
Sex			5928	
Female	14	36.8		
Male	24	63.2		
Organ transplanted				
Kidney	19	50	1641	11.6
Liver	9	26.3	2400	3.8
Heart	1	2.6	268	3.7
Pancreas	1	2.6	64	15.6
Lung	5	13.1	160	31.3
Bone marrow	2	5.2	1219	1.6
Kidney + heart	2	2.6	176	5.7
Immunosuppressant drug				
Tacrolimus	12	31.6		
Tacrolimus + others	15	39.5		
Sirolimus	6	15.8		
Others	3	7.9		
Serum PCR for EBV				
Negative	14	45.2		
Positive	17	54.8		
Infiltration of the bone marrow				
Negative	31	88.6		
Positive	4	11.4		
Histology (monomorphic)	34	89.47		
Hodgkin lymphoma	3	7.9		
Non‐Hodgkin lymphoma	31	92.1		
Histological subtypes (non‐Hodgkin)	31			
Anaplastic	2	6.45		
Diffuse large B‐cell lymphoma	25	80.60		
Burkitt	4	12.90		
Plasmacytic hyperplasia	1	2.63		
Polymorphic	3	7.89		
EBV PCR (biopsy specimens)				
Negative	14	37.8		
Positive	23	62.2		
Stage				
I	10	28.6		
II	2	5.7		
III	1	2.9		
IV	22	62.9		
Chemotherapy, first line				
R‐CHOP‐like	15	42.9		
Rituximab	8	21.0		
Rituximab + associations	4	11.4		
Others	9	25.7		
Chemotherapy, second line				
Rituximab	7	20		
Others	28	80		
Response to treatment				
No	9	25.7		
Yes	26	74.3		
Death				
No	22	57.9		
Yes	16	42.1		
Total	38	100		
Age (years)	Median	IQR		
	53	31–65		
Laboratory characteristics	Mean	SD		
Lactate dehydrogenase (U/L)	985.32	946.61		
Albumin (g/L)	3.26	0.61		
Beta‐2 microglobulin (mg/L)	7.82	9.09		
Hemoglobin (g/dL)	10.54	2.24		

Table 1 lists patients’ histopathology results. Histologically, 89.5% of cases consisted of monomorphic PTLD, predominantly diffuse large B‐cell lymphoma (DLBCL) and Burkitt lymphoma. EBV DNA was positive in 54.8% of serum samples and 62.2% of tissue biopsies. Extranodal involvement was found in 68% of cases, particularly in the gastrointestinal tract (44.7%) and central nervous system (13.1%).

All treated patients underwent immunosuppression reduction, with 35 receiving anti‐CD20 therapy, either as monotherapy or combined with chemotherapy. Rituximab plus cyclophosphamide, doxorubicin, Oncovin, and prednisone (R‐CHOP) was the most common first‐line regimen (*n* = 15), followed by rituximab alone (*n* = 8). Second‐line treatment was required in 7 refractory cases, and one patient underwent CAR‐T therapy following relapse.

Triple immunosuppression was used in 9 patients (23.68%), with 6 of these (66.6%) using tacrolimus + prednisone + mycophenolate mofetil (MMF). In addition, 12 individuals (31.6%) used tacrolimus monotherapy, 6 (15.8%) used tacrolimus + prednisone, and 6 (15.8%) used sirolimus.

The predictors of overall survival (OS) are listed in Table 2. Multivariate analysis revealed age and treatment response to be independent predictors of OS (Table 3). Patients at Ann Arbor Stage I/II had a 5‐year OS of 80% compared to 30% for those at Stage III/IV. Patients who responded to treatment had an OS > 80% compared to < 20% for nonresponders (Figure 2). The overall mortality rate was 42%, with death occurring primarily from sepsis and disease progression.

**Table 2:** Factors associated with survival in the cohort of patients diagnosed with posttransplant lymphoproliferative disorder (PTLD).

Factor	Mean survival time (months)	95% CI		HR	95% CI		Death	Total *N*	%	*p* value
		Lower	Upper		Lower	Upper				
Age (years)				1.03	1.00	1.05				**0.042** ^∗^
Sex										0.089
Female	92.0	61.9	122.2	1.00			4	14	28.6	
Male	28.3	17.2	39.5	2.62	0.83	8.24	12	24	50.0	
Immunosuppressant drug										0.652
Tacrolimus	54.8	32.0	77.6	1.00			5	14	35.7	
Tacrolimus + others	68.0	33.1	102.8	1.01	0.30	3.40	6	15	40.0	
Sirolimus	44.8	19.5	70.1	0.90	0.21	3.91	3	6	50.0	
Others	14.3	10.3	18.2	2.54	0.48	13.29	2	3	66.7	
Serum PCR for EBV										0.864
Negative	28.8	18.0	39.5	1.00			6	14	42.9	
Positive	60.2	30.4	90.0	1.10	0.39	3.09	9	17	52.9	
Infiltration of the bone marrow										0.378
Negative	44.2	27.9	60.5	1.00			13	31	41.9	
Positive	38.8	0.0	90.0	1.75	0.50	6.17	3	4	75.0	
Histology										0.680
Hodgkin lymphoma	29.3	26.8	31.8	1.00			1	3	33.3	
Non‐Hodgkin lymphoma	64.8	41.8	87.7	1.53	0.20	11.74	15	35	42.9	
EBV PCR (biopsy specimen)										0.765
Negative	40.7	22.2	59.2	1.00			6	14	42.9	
Positive	64.6	36.1	93.1	1.17	0.42	3.22	10	23	43.5	
Staging										**0.050**
I/II	108.1	81.8	134.4	1.00			2	12	16.7	
III/IV	36.3	20.0	52.6	3.98	0.90	17.68	13	23	56.5	
First‐line chemotherapy										0.093
R‐CHOP‐like	43.6	21.4	65.8	1.00			7	15	46.7	
Rituximab	110.6	77.5	143.8	0.21	0.03	1.68	1	7	14.3	
Rituximab + associations	11.7	2.5	20.8	2.75	0.67	11.28	3	4	75.0	
Others	26.9	15.9	37.9	1.02	0.30	3.54	4	9	44.4	
Second‐line chemotherapy										0.063
Rituximab	110.6	77.5	143.8	1.00			1	7	14.3	
Others	36.6	20.9	52.4	5.57	0.73	42.68	14	28	50.0	
Treatment response										**0.007**
No	21.5	4.3	38.8	1.00			7	9	77.8	
Yes	83.7	56.6	110.8	0.26	0.09	0.74	7	26	26.9	
Lactate dehydrogenase (× 100)				1.04	1.00	1.09				0.075^∗^
Albumin				0.63	0.28	1.39				0.250^∗^
Beta‐2 microglobulin				1.04	0.99	1.11				0.146^∗^
Hemoglobin				1.00	0.81	1.25				0.979^∗^
Interval between organ transplant and PTLD (months)			1.01	1.00	1.02				0.269^∗^	
Number of chemotherapy cycles				0.62	0.46	0.84				**0.002** ^∗^
Transplanted organ^†^										0.214
Kidney	47.8	27.6	68.0	1.00			8	18	44.4	
Liver	113.8	86.1	141.5	0.25	0.03	2.04	1	9	11.1	
Lung	19.5	5.0	34.0	1.62	0.43	6.13	3	5	60.0	
Overall	**64.3**	**41.9**	**86.7**				**16**	**38**	**42.1**	

*Note:* The bold values represent important points for attention.

Abbreviation: HR = hazard ratio.

^†^Only for the most commonly transplanted organs; log‐rank test.

^∗^Univariate Cox regression.

**Table 3:** Multivariate Cox regression analysis for survival in the cohort of patients diagnosed with posttransplant lymphoproliferative disorder (PTLD).

Factor	HR	95% CI		Death
		Lower	Upper	
Age (years)	1.04	1.00	1.08	**0.04**
Stage (III/IV)	6.62	0.72	60.60	0.1
Treatment response	0.11	0.02	0.55	**0.01**

*Note:* The bold values represent important points for attention.

Abbreviation: HR = hazard ratio.

Figure 2(a) Analysis of overall survival as estimated using Kaplan–Meier curves according to the Ann Arbor Stage. (b) Analysis of overall survival estimated using Kaplan–Meier curves according to response to treatment. Time zero in the graph corresponds to the date of patient referral.

**Figure:**
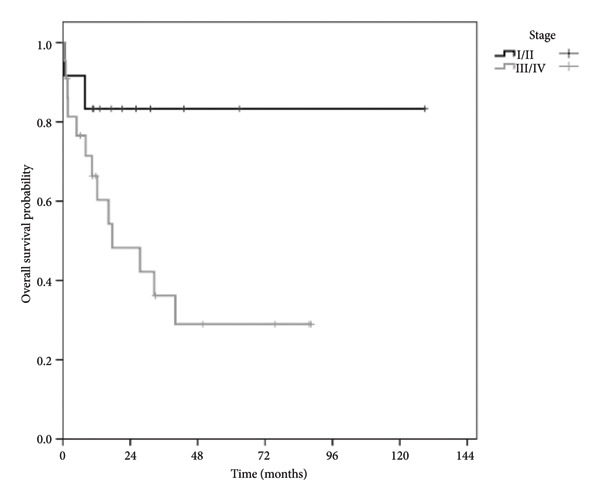
(a)

**Figure:**
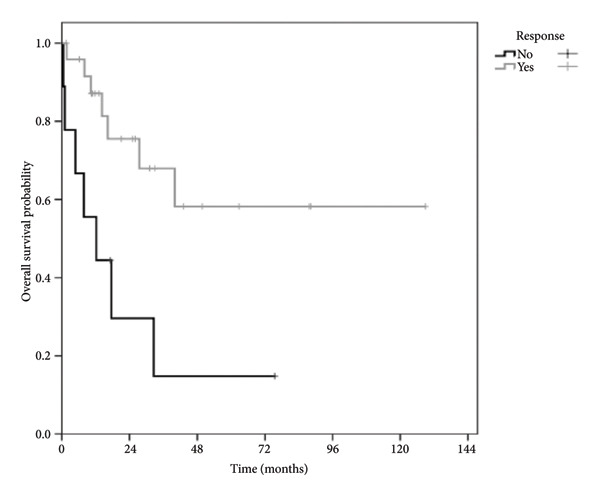
(b)

## 4. Discussion

Evaluation of this 24 year, single‐center cohort showed the incidence of PTLD in Brazilian transplant recipients to be in line with global data, despite the different epidemiology of EBV in Brazil. Janeela et al.​ [5] reported an incidence of PTLD of 1%–10% in adults, with a 5‐year cumulative incidence rate varying according to the organ transplanted: bowel (20%), lung (3%–10%), heart (2%–8%), liver (1%–5.5%), pancreas (0.5%–5.0%), and kidney (0.8%–2.5%) [3]. In the present study, also conducted with an adult sample, the incidence of PTLD was 0.6%. Median age was 53 years, and 63% of the patients were male. Lung transplant recipients were those most affected, mirroring findings in high‐income countries [13].

Although PTLD is a serious complication of organ transplantation, it is rare and has generally been evaluated in smaller, less robust studies [13]. This paucity of data hampers the standardization of diagnosis and treatment for this group of patients. The risk factors for PTLD include the amount of lymphoid tissue in the graft, the transplant recipient’s age and sex, the number of rejection episodes, primary EBV infection, the type of immunosuppressive drugs used, and the degree of compatibility between donor and recipient [13, 14].

A key strength of this study is the comparative analysis between PTLD and HIV‐associated lymphomas. Both groups share immunosuppression as a driving factor for lymphomagenesis, and disease stages tend to be advanced at diagnosis. In this intrainstitutional comparison, PTLD and HIV‐related lymphoma patients had similar demographic and histopathologic profiles, with high rates of extranodal involvement and EBV positivity in tissue. However, lung and multivisceral involvement was more common in the PTLD cases, likely due to organ‐specific immunosuppression protocols. Despite these differences, OS did not differ significantly between the groups [2]. These findings underscore the shared pathogenic mechanisms and clinical challenges in managing immunodeficiency‐associated lymphomas, reinforcing the value of cross‐cohort comparisons in shaping treatment approaches. Grulich et al. [8] conducted a meta‐analysis comparing the incidence of cancer in immunosuppressed HIV/AIDS patients and in patients who were immunosuppressed following organ transplant. The incidence of lymphomas, either Hodgkin’s or non‐Hodgkin’s, was higher in these two groups of patients, with no mention of any difference in survival (OS) between the two groups (PTLD: 0.4; HIV: 0.5; *p* = 0.255) [2, 8].

A notable feature of this cohort is the high frequency of monomorphic PTLD and significant extranodal involvement, particularly at sites in the gastrointestinal tract and central nervous system. Kremer et al. [16] reported gastrointestinal symptoms in 22.5% of the patients with PTLD and Ann Arbor Stage III/IV in 44%. Wudhikarn et al. [14] evaluated 32 cases of monomorphic PTLD, 28 (87.5%) of whom were classified as having DLBCL, 1 (3.125%) Burkitt lymphoma, 1 (3.125%) anaplastic lymphoma, and 2 (6.25%) undetermined. While EBV positivity in serum was moderate (54.8%), the majority of biopsies were EBV‐positive, reaffirming the virus’s etiological role. In the study conducted by Kremer et al., 89% of patients were positive for serum EBV DNA [16]. Interestingly, the dominant EBV genotype and LMP1 polymorphisms in Brazil did not appear to increase the incidence of the disease but may affect its severity.

Comparisons with HIV‐associated lymphomas revealed clinical and virological similarities, including advanced‐stage disease and EBV positivity. However, lung and multivisceral involvement was more common in PTLD cases, possibly reflecting the underlying immunosuppressive regimens [8]. Serraino et al. [17] reported a significantly elevated risk of lung cancer in heart transplant recipients (SIR = 2.8) and a risk of borderline statistical significance in HIV‐positive people (95% CI: 0.9–2.8). Immune depression entails a two‐fold increase in the overall risk of cancer, mainly related to cancers associated with a viral etiology.

This study highlights the need for standardized PTLD surveillance in Latin America and suggests that future research should explore regional viral–genetic interactions, including LMP1 variants [3, 4]. The inclusion of advanced therapies such as CAR‐T in this setting emphasizes the evolving treatment capacity, even in middle‐income countries. These results are coherent, and the incidence of PTLD in this Brazilian cohort aligns with that reported in developed countries, despite differences in EBV prevalence and subtypes. Limitations include its retrospective, single‐center design; incomplete historical records, particularly regarding performance status; lack of molecular EBV typing; and the inability to perform multivariable adjustment for transplant type due to the small sample size. Nonetheless, the long duration of the investigation reinforces its relevance.

## 5. Conclusion

The findings of the present study confirm that the incidence of PTLD in Brazil mirrors that of developed nations, supporting the effectiveness of organ transplant and oncologic care. EBV detection in biopsies reinforces its pathogenetic role. The lung was the transplanted organ most associated with PTLD, and monomorphic histology predominated. Age and treatment response remain key prognostic indicators. These insights underscore the importance of integrating local epidemiology with global evidence to refine PTLD management strategies.

## Conflicts of Interest

The authors declare no conflicts of interest.

## Funding

This study received no external funding.

## Data Availability

Data will not be shared publicly due to the possibility of patient identification.
